# Influences of Thermal Stress During Three Weeks Before Market Age on Histology and Expression of Genes Associated With Adipose Infiltration and Inflammation in Commercial Broilers, Native Chickens, and Crossbreeds

**DOI:** 10.3389/fphys.2022.858735

**Published:** 2022-04-12

**Authors:** Yuwares Malila, Pornnicha Sanpinit, Wilawan Thongda, Anuwat Jandamook, Yanee Srimarut, Yupin Phasuk, Sajee Kunhareang

**Affiliations:** ^1^ National Center for Genetic Engineering and Biotechnology (BIOTEC), Thailand Science Park, Pathum Thani, Thailand; ^2^ Center of Excellence for Shrimp Molecular Biology and Biotechnology (CENTEX Shrimp), Faculty of Science, Mahidol University, Bangkok, Thailand; ^3^ Department of Animal Science, Faculty of Agriculture, Khon Kaen University, Khon Kaen, Thailand

**Keywords:** chicken, droplet digital polymerase chain reaction, fat accumulation, immune cell infiltration, heat stress

## Abstract

The objectives of this study were to examine the effects of cyclic thermal stress on histological characteristics of breast muscle and gene expression regarding adipose infiltration and inflammation in breast muscles collected from different breeds of chickens. The birds, from commercial broilers (CB, Ross 308, 3 weeks), native (NT, 100% Thai native Chee, 9 weeks), H75 (crossbred; 75% broiler and 25% NT, 5 weeks), and H50 (crossbred; 50% broiler and 50% NT, 7 weeks), were equally assigned into control or treatment groups. The control samples were reared under a constant temperature of 26 ± 1°C, while the treatment groups were exposed to 35 ± 1°C (6 h per day). After a 20-day thermal challenge, 12 male birds per treatment group were randomly collected for determination of live body weight, breast weight, numbers of growth-related myopathies, and breast meat chemical composition. Histological lesions were evaluated in the pectoralis major muscle immediately collected within 20 min postmortem based on hematoxylin and eosin staining. The results indicated that despite interaction between thermal stress and breed effects, thermal challenge significantly reduced feed intake, live body weight, and breast weight of the birds and increased moisture content in breast meat (*p* < 0.05). An interaction between the two main factors was found for protein content (*p* < 0.05) for which control CB showed less protein than the other groups. Heat stress decreased histological scores for adipose infiltration in CB (*p* < 0.05), but it did not significantly influence such scores in the other groups. CB received histological scores for adipose tissue at greater extent than those for the other groups. Differential absolute abundance of *CD36*, *FABP4*, *LITAF*, *PDGFRA*, *PLIN1*, *PPARG*, *POSTN*, *SCD1*, and *TGFB1* in the muscle samples well-agreed with the trend of histological scores, suggesting potential involvement of dysregulated fibro-adipogenic progenitors together with imbalanced lipid storage and utilization in the breast muscle. The findings demonstrated that the cyclic thermal challenge restricted growth performance and breast mass of the birds, but such effects attenuated infiltration of adipose tissue and inflammatory cells in the CB breast muscle.

## Introduction

An increase in average environmental temperatures has urgently raised global attention as it inevitably impacts the well-being of lives on Earth. For chickens, their physiological response to thermal challenge depends on several factors, including breed, size, and age (Lu et al., 2007; Awad et al., 2020; Malila et al., 2021a). Commercial broilers, meat-type chickens, exhibit more susceptibility to thermal stress than their ancestors (Tabler et al., 2020) and other slow-growing strains (Washburn et al., 1980; Aengwanich, 2007; Malila et al., 2021a). This has been considered a negative consequence of an artificial breeding selection program focused on production performance and massive mass to meet high consumer demand. Development of thermoregulatory systems of broilers does not complement rapid growth of the birds (Havenstein et al., 2003). An ambient temperature above 30°C is considered sufficient to induce stress among the birds (Azad et al., 2010; Adu-Asiamah et al., 2021). The negative impacts of thermal challenge include diminished growth performance (Azad et al., 2010; Quinteiro-Filho et al., 2010; He et al., 2018; Aslam et al., 2021), increased mortality (Al-Fataftah and Abdelqader, 2014), compromised gut health, (Quinteiro-Filho et al., 2010; Awad et al., 2020), and deviated meat quality (Sandercock et al., 2001; Lu et al., 2017; Malila et al., 2021a). A recent meta-analysis performed by Andretta et al. (2021) highlighted that broilers with increasing age and weight become more sensitive to heat as their heat dissipation area becomes smaller. Their findings also indicated that heat stress negatively affects broilers over 21 days of age at a greater extent than the birds at an initial growing phase.

In regard to altered meat quality, increased fat along with decreased protein content was frequently reported in the meat yielded from chickens exposed to heat stress (Akşit et al., 2006; Lu et al., 2007; Zhang et al., 2012). The actual molecular etiology of the changes remained not fully comprehended. An increased level of serum corticosterone was observed in heat-stressed chickens and further linked with induced insulin resistance, stimulated lipid synthesis, and fat accumulation in abdominal, cervical, and thigh adipose tissues (Quinteiro-Filho et al., 2010; Xu et al., 2018). Serum corticosterone might also activate protein breakdown in the skeletal muscle of stressed animals (Scanes, 2016). Lu et al. (2019) reported widespread deposition of lipid droplets inside the liver of Arbor Acres broilers exposed to 14-day constant heat stress (32°C). They found that in the stressed animals, both apolipoprotein B gene and protein levels in the chicken liver and plasma corticosterone concentration remained unchanged, suggesting the disrupted transportation of the excessive triglycerides from the liver in the broilers exposed to chronic heat stress. Recently, Adu-Asiamah et al. (2021) analyzed histopathological characteristics of 56-day-old Chinese broiler chickens exposed to acute heat stress (35°C) for 8 h and reported the extent of tissue damage in the liver, spleen, breast, and leg muscles. In addition, experiments conducted using turkey’ pectoralis major muscle satellite cells showed that temperature extremes altered the adipocyte-like properties of the satellite cells isolated from fast-growing and non-selected random-bred control lines (Clark et al., 2017).

The cyclic thermal stress, resembling the daily rise of temperature in the tropical region or in the summer of the temperate zone, exerted adverse impacts on growth performance of commercial broilers, although the impact was at a lesser extent than that of the constant heat challenge (Souza et al., 2016; Aslam et al., 2021). Under cyclic stress, commercial broilers are partly able to adjust to the stressed environment and have compensatory gain during the non-stressed period (Aengwanich, 2007; Andretta et al., 2021). Still, reduced growth performance and less meat yield, attributed to an exposure to cyclic heat stress, were consistently reported among previous studies (Quinteiro-Filho et al., 2010; Shao et al., 2019; El-Tarabany et al., 2021). Heat stress may exert a negative impact on meat quality through oxidative damages (Lu et al., 2017). This could impair the muscle regeneration process and lead to muscle abnormalities, including emerging myopathies known as white striping (WS) and wooden breast (WB), among the chickens exposed to thermal challenge (Aslam et al., 2021). Several studies indicated chemical changes, that is, reduced protein and increased fat content in chicken meat affected with those abnormalities (Kuttappan et al., 2012; Petracci et al., 2014; Malila et al., 2018; Thanatsang et al., 2020). Overall, the incidence could not only affect economic returns of broilers but also potentially dilute nutritional properties of chicken breast meat, which is generally recognized as an inexpensive source of food protein in several regions.

The objectives of this study were to examine the effects of cyclic thermal stress, mimicking the rise of temperature during the day in tropical regions, on histological characteristics of the breast (pectoralis major) muscle of chickens from different breeds. Absolute transcript abundance of associated genes was also evaluated to provide insights at molecular levels. The chickens included commercial broiler (CB, Ross 308), native (NT, 100% Thai native Chee), H75 (crossbred; 75% broiler and 25% NT), and H50 (crossbred; 50% broiler and 50% NT). We focused on the exposure of the challenge during the last 3 weeks before the specific market ages of each breed.

## Materials and Methods

### Experimental Design, Animals, and Management

All chickens, including commercial broiler (CB, Ross 308), native (NT, 100% Thai native Chee), H75 (crossbred; 75% broiler and 25% NT), and H50 (crossbred; 50% broiler and 50% NT), were raised and maintained under an environmentally controlled poultry facility of the Department of Animal Science, Faculty of Agriculture, Khon Kaen University (Khon Kaen, Thailand) following the standard practice for commercial broilers. The birds were housed in floor pens (1.3 m × 2.0 m) with rice hulls provided as bedding materials and received standard commercial broiler diets (Table 1). Feed and water were provided *ad libitum*. A routine vaccination program against coccidiosis, infectious bronchitis virus, and Newcastle disease was applied.

**TABLE 1:** Chemical composition of standard commercial broiler diets.

Nutrient	Starting phase (1 day–21 days)	Growing phase (22 days to Market age)
Metabolizable energy (kcal/kg)	3,100	3,200
Moisture (%)	13	13
Crude protein (%)	21	20
Crude fiber (%)	5	5
Fat (%)	4	4

Three weeks prior to their specific market ages, the chickens of each strain were randomly divided into two groups: control and stressed treatment. The control (4 replications/group, 12–13 birds/replication, 5 birds/m^2^) group was kept at a constant temperature of 26 ± 1°C, while the treatment groups (4 replications/group, 12–13 birds/replication, 5 birds/m^2^) were exposed to 35 ± 1°C during 10:00 a.m. to 4:00 p.m., accounting for 6 h daily. The thermal challenge period was carried on for 20 days.

Upon completion of the thermal challenge, a total of 96 male chickens (*n* = 12 per group, 3 birds per pen replicate) were proceeded to the slaughtering process with 12-h fasting prior to the slaughter. The right side of the breast was immediately removed from the carcass and monitored for occurrences (presence or absence) of white striping (WS) and wooden breast (WB) abnormalities based on the criteria previously described by Malila et al. (2018) and Tijare et al. (2016), respectively. The evaluation was carried out by one fully trained staff to minimize the variation. The muscle samples were excised, oriented along the muscle fiber, from the cranial portion (approximately 1 cm deep from the ventral surface) of the muscle, and cut into 0.5 cm^3^ cubes. Half of the muscle specimen was then snap-frozen in liquid nitrogen within 20 min postmortem and stored at −80°C until RNA isolation, whereas the other half was fixed within 10% buffered formalin fixative (pH 7.0) and stored at 4°C until histological evaluation.

The left side of the breast was collected, weighed, packed in a plastic bag, kept on ice during samples collection, and subsequently stored at refrigerated temperature until it reached 24 h postmortem. The samples were then ground and stored at −20°C until the analyses of chemical composition were performed.

### Proximate Composition

Chemical compositions, including moisture, protein, fat, ash, and carbohydrate, of the samples were determined following the standard methods of AOAC (2016). In brief, moisture content was analyzed based on weight loss upon drying the samples at 105°C. Crude protein was determined following a Dumas combustion principle, and the conversion factor of 6.25 was used for calculation (AOAC 990.03). Crude fat was extracted from the breast samples with petroleum ether by using a Soxtherm (model SOX416, C. Gerhardt GmbH & Co. KG, Königswinter, Germany) following a Soxhlet method. Ash content was examined based on an incineration of the samples at 600°C. The analyses were performed in two technical replicates.

### Histological Evaluation and Scoring

The muscle specimen stored in 10% buffered formalin was dehydrated in ethanol solutions with a serial concentration of 70, 80, 95% (repeated at this step three times), and 100%. The specimen was then embedded in paraffin and cross-sectioned into 5-µm sections. The tissue section slides were stained with hematoxylin and eosin (H&E staining). The slide section was examined under a bright-field microscope (Olympus, Tokyo, Japan) equipped with a digital camera (Olympus DP73 Microscope Digital Camera, Tokyo, Japan) and visualized using Olympus cellSens software (Olympus, Tokyo, Japan). The histopathological characteristics, including inflammation and adipose infiltration, were scored based on the criteria described by Prisco et al. (2021). In brief, inflammation was assessed based on the number of inflammatory cells as follows: score 0 = no infiltrated cells; score 1 = 5 to 25 cells per field; score 2 = 26 to 50 cells per field; and score 3 = more than 50 cells per field. As for adipose tissue infiltration: score 0 = no appearance of adipose tissue; score 1 = less than 10% of the skeletal muscle area was infiltrated by adipose tissue; score 2 = 10 to 20% infiltration; and score 3 = more than 20% infiltration. A total of ten fields at ×200 magnification (×20 for objective lens and ×10 for eyepiece lens) for each sample were selected for the evaluation. The scores for each muscle sample were an average score calculated from the ten fields.

### Total RNA Isolation and cDNA Synthesis

Total RNA was isolated from the frozen breast muscle tissues using TriReagent (Molecular Research Center, Inc., Cincinnati, OH, United States) following the manufacturer’s protocol. Contaminated DNA was removed by incubating the isolated total RNA with RNase-free DNase I (Thermo Fisher Scientific, Rockford, IL, United States), according to the company’s instruction. The total RNA samples were then purified using a column-based ReliaPrep™ RNA clean-up and concentration system (Promega Corporation, Madison, WI, United States). The quantity and quality of total RNA were determined using a Nanodrop spectrophotometer (Thermo Fisher Scientific) and a fragment analyzer system (model 5300, Agilent), respectively. Only RNA samples exhibiting RNA integrity number greater than 7.0 were used for cDNA synthesis. Subsequently, total RNA (1.5 µg) was reverse-transcribed into cDNA using oligo(dT) as a primer and an ImProm-II™ reverse transcription system (Promega Corporation). The amount of the synthesized cDNA was determined using a Nanodrop spectrophotometer (Thermo Fisher Scientific).

### Primers and Droplet Digital Polymerase Chain Reaction

The absolute expressions of 13 target genes (Table 2) associated with lipid metabolism and muscle injury were evaluated using an EVAGREEN droplet digital polymerase chain reaction (ddPCR) assay. The primers were designed using Primer-BLAST (https://www.ncbi.nlm.nih.gov/tools/primer-blast/). To confirm primer specificity, PCR mixture, containing 1X EvaGreen supermix (Bio-Rad Laboratories, Inc., Hercules, CA, United States), 0.25 µM of each forward and reverse primer, and the cDNA template (Table 2), was prepared (Table 2) and performed using a thermocycler with conditions set as 95°C for 5 min; 40 cycles of 95°C for 30 s, 60°C for 1 min, 4°C for 5 min, and 90°C for 5 min. Primer specificity was then confirmed by the presence of a single band of amplicon products that corresponded to the correct molecular weight on a 2% agarose gel.

**TABLE 2:** Primers.

NCBI accession number	Gene ID	Gene annotation	Sequence (5′ → 3′)	Amplicon length (bp)	Template amount (ng)	Annealing temperature (°C)
NM_206,991.1	*ADIPOQ*	Adiponectin	F: AGC​AGA​ACC​ACT​ACG​ACA​GC	166	10	60
—	—	—	R: ACG​TTG​TTC​TCC​TGG​AAC​TGG	—	—	—
NM_001,030,731	*CD36*	CD36 molecule	F: TAC​CAG​ACC​AGT​AAG​ACC​GTG​AAG​G	156	25	60
—	—	—	R: AAT​GTC​TAG​GAC​TCC​AGC​CAG​TGT	—	—	—
NM_204,290.1	*FABP4*	Fatty acid–binding protein 4	F: TAT​GAA​AGA​GCT​GGG​TGT​GG	168	10	60
—	—	—	R: GCT​GTG​GTC​TCA​TCA​AAC​TC	—	—	—
NM_204,267.1	*LITAF*	Lipopolysaccharide-induced tumor necrosis factor-alpha factor	F: ACT​ATC​CTC​ACC​CCT​ACC​CTG​TC	95	25	60
			R: TGT​TGG​CAT​AGG​CTG​TCC​TG			
XM_040,666,999.1	*LPIN1*	Lipin 1	F: CCT​TTC​ACT​GTA​ATG​CTG​GT	173	10	60
—	—	—	R: TGG​AGT​GGT​ATG​GTC​ATC​AG	—	—	—
NM_205,282.1	*LPL*	Lipoprotein lipase	F: AGG​AGA​AGA​GGC​AGC​AAT​A	222	10	60
—	—	—	R: AAA​GCC​AGC​AGC​AGA​TAA​G	—	—	—
NM_001,030,363.1	*MYF5*	Myogenic factor 5	F: TGA​ACC​AAG​CAT​TCG​AGA​CC	141	10	60
			R: AGT​AGT​TCT​CCA​CCT​GTT​CCC​T			
NM_204,749.2	*PDGFRA*	Platelet-derived growth factor receptor, alpha	F: AAG​AGA​GTG​CCA​TTG​AAA​CCG	155	10	60
			R: GCA​GTT​AGA​AGG​TGT​CTG​GGA​T			
NM_001,127,439.1	*PLIN1*	Perilipin 1	F: GCCAAGGAGAACGTGCT	142	10	60
—	—	—	R: TCA​CTC​CCT​GCT​CAT​AGA​CC	—	—	—
NM_001,001,460.1	*PPARG*	Peroxisome proliferator activated	F: CCA​GCG​ACA​TCG​ACC​AGT​TA	182	100	60
—	—	Receptor, gamma	R: TCC​CAT​CCT​TAA​AGA​GTT​CA	—	—	—
NM_001,030,541.1	*POSTN*	Periostin	F: GCC​TGG​TGT​GAC​AAA​CAT​CC	119	10	60
—	—	—	R: TGG​TTG​CCA​TGA​GAT​CAG​GTT	—	—	—
NM_204,890.1	*SCD1*	Stearoyl-CoA desaturase 1	F: GGC​TGA​CAA​AGT​GGT​GAT​G	137	50	60
—	—	—	R: GGATGGCTGGAATGAAGA	—	—	—
NM_001,318,456.1	*TGFB1*	Transforming growth factor beta 1	F: GAC​GAT​GAG​TGG​CTC​TCC​TTC	195	10	60
			R: GTG​CTT​CTT​GGC​AAT​GCT​CT			

To perform ddPCR, the ddPCR reaction mixture consisting of 1X EvaGreen supermix (Bio-Rad Laboratories, Inc., Hercules, CA, United States), 0.25 µM of each forward and reverse primer, and the cDNA template (Table 2), was set up in a sterile microcentrifuge tube. The template was replaced by an equal volume of nuclease-free water for no template control. The ddPCR reaction mixture (20 μL) was then mixed with Evagreen generator oil (Bio-Rad Laboratories, Inc., Hercules, CA, United States) using a QX100™ droplet generator (Bio-Rad Laboratories, Inc.), according to the company’s protocol. The generated droplets (40 μL) were subsequently transferred into a 96-well plate. The plate was then heat-sealed with an aluminum foil cover. The target genes in the samples were amplified in a thermocycler (model T100™, Bio-Rad Laboratories, Inc.) with a condition set as follows: 95°C for 5 min; 40 cycles at 95°C for 30 s, 60°C for 1 min, and 4°C for 5 min; and 90°C for 5 min. Afterward, the fluorescent signal intensity of the droplets was measured using a QX200™ droplet reader (Bio-Rad Laboratories, Inc.). The number of positive and negative droplets was automatically counted by QuantaSoft™ software (Bio-Rad Laboratories, Inc.), and transcript abundance was expressed as copies per 20-µL reaction.

### Statistical Analysis

A statistical analysis was performed using the R package version 3.4.3. The significant level for all statistical analyses was set at *α* = 0.05. The effects of thermal challenge and chicken breeds, as the main effects, were determined according to a 2 × 4 factorial analysis of variance (ANOVA). The mean differences were assessed using Duncan’s new multiple range test. Prior to ANOVA analysis, the assumptions of normality and homogeneity of variance were examined using the Shapiro–Wilk normality test and Bartlett test, respectively. The data set that did not follow the assumptions was transformed using a function *varIdent* from the library nlme of the R package.

Pearson’s correlation coefficient was calculated to define the correlation among histological scores and absolute expression of the tested genes. In this case, average histological scores from 10 observed microscopic fields and absolute transcript abundance (in the unit of copy number per 20 µL reaction) were included in the test. As for the relationship between growth-related myopathies and gene expression level, this categorical variable was labeled as 1 (presence) or 0 (absence) before the data were submitted to Spearman’s rank correlation test.

## Results and Discussion

### Feed Intake, Live Body Weight, Breast Weight, Chemical Composition, and Cases of Growth-Related Myopathies

Table 3 shows feed intake (during the 20-day thermal stress), live body weight, breast weight, and breast yield of the tested chickens as affected by the thermal challenge. Focusing on the main effects, the heat condition reduced feed intake, live body weight, and breast weight (*p* < 0.05) but not breast yield (*p* ≥ 0.05). Live body weight of the stressed CB, stressed H50, and stressed NT was approximately 13, 11, and 12%, respectively, less than their control counterparts. The current stress condition on reduced breast weight was more pronounced in CB as the breast weight was reduced to 28, 8, and 13% in stressed CB, stressed H50, and stressed NT, respectively, in comparison to their control counterparts. The reduced body weight and breast weight due to the thermal challenge in those breeds well agreed with that observed in previous studies (Sandercock et al., 2001; Duangjinda et al., 2017; Shao et al., 2019; Emami et al., 2021). Duangjinda et al. (2017) compared growth performance of commercial broilers and Thai indigenous chickens under either thermoneutral condition (26°C) or heat challenge (36–38°C, 6 h daily) for 3 weeks prior to market age and reported decreased growth performance for broilers but not in the indigenous birds. Shao et al. (2019) reported reduced body weight, average daily gain, and average feed intake in yellow-feathered broilers exposed to cyclic temperature of 35°C (8 h daily) for 7 days compared to the birds reared under thermal neutral condition (23°C). Using similar thermal challenge conditions (cyclic temperature of 35°C, 8 h daily vs. control condition of 23 °C), Emami et al. (2021) observed a significant decrease in body weight of broilers exposed to the stress during the age of 29–42 days. The decreased production performance has been shown to be associated with restricted feed intake upon exposure to thermal challenge (Souza et al., 2016). In this study, the difference in feed intake during the 20-day challenge was observed only between the control and stressed H50 (*p* < 0.05). The previous meta-analysis conducted by Andretta et al. (2021) found that under cyclic thermal stress, commercial broilers at the age of 21 days or older could sometime compensate their intake during the cooling period. The lack of thermal effects on growth and meat yield of H75 and NT suggested better heat tolerance and better adaptation of both strains over CB and H50 (Aengwanich, 2007).

**TABLE 3:** Live body weight at slaughter age, breast weight, and breast yield (%) of the chicken samples.

Sample		Feed intake^1^ (g/bird/day)	Live body weight (g)	Breast weight (g)	Breast yield^2^ (%)
CB (42d)^3^	Control	98.3 ± 11.6	1,962.5 ± 188.8	164.8 ± 43.6	8.3 ± 1.5
—	Treatment	85.6 ± 3.0	1,695.0 ± 106.3	119.3 ± 13.5	7.0 ± 0.4
H75 (56d)	Control	85.0 ± 3.8	1,575.0 ± 125.8	82.3 ± 19.1	5.2 ± 1.0
—	Treatment	88.2 ± 13.2	1,576.3 ± 115.9	88.8 ± 15.9	5.6 ± 0.8
H50 (70d)	Control	94.4 ± 8.6	2,150.0 ± 122.5	132.8 ± 15.7	6.2 ± 0.7
—	Treatment	79.8 ± 6.3	1,905.0 ± 85.4	121.5 ± 10.1	6.4 ± 0.4
NT (84d)	Control	64.4 ± 3.8	1,345.0 ± 68.1	60.5 ± 8.2	4.5 ± 0.4
—	Treatment	63.9 ± 8.2	1,185.0 ± 54.5	52.5 ± 4.4	4.4 ± 0.3
Main effects	—	—	—	—	—
Stress	Control	86.9 ± 15.05	1,758.1 ± 347.5	110.1 ± 48.1	6.8 ± 1.9
—	Treatment	78.8 ± 11.54	1,590.3 ± 283.2	95.5 ± 30.8	6.5 ± 1.2
Breed	CB	92.0^a^ ± 10.4	1,828.8^b^ ± 201.4	142.0^a^ ± 38.5	8.4^a^ ± 1.4
—	H75	86.4^a^ ± 8.3	1,575.6^c^ ± 112.0	85.5^b^ ± 16.7	6.1^b^ ± 1.0
—	H50	87.1^a^ ± 10.5	2,027.5^a^ ± 163.4	127.1^a^ ± 13.6	6.9 ^b^ ± 0.6
—	NT	64.2^b^ ± 5.7	1,265^d^ ± 102.8	56.5^c^ ± 7.5	5.0^c^ ± 0.4
*p*-value	—	—	—	—	—
Stress	—	0.03	0.0004	0.04	0.34
Breed	—	<0.0001	<0.0001	<0.0001	<0.0001
Interaction	—	0.12	0.11	0.09	0.15

Mean ± standard deviation (*n* = 12).

1 Feed intake during the last 3 weeks before specific market age.

2 Breast yield was expressed in percentage as breast weight relative to live body weight.

3 Slaughter age (days) of each chicken strain.

^a, b, c, d^ Significant differences due to different breeds (*p* < 0.05).

**FIGURE 1:**
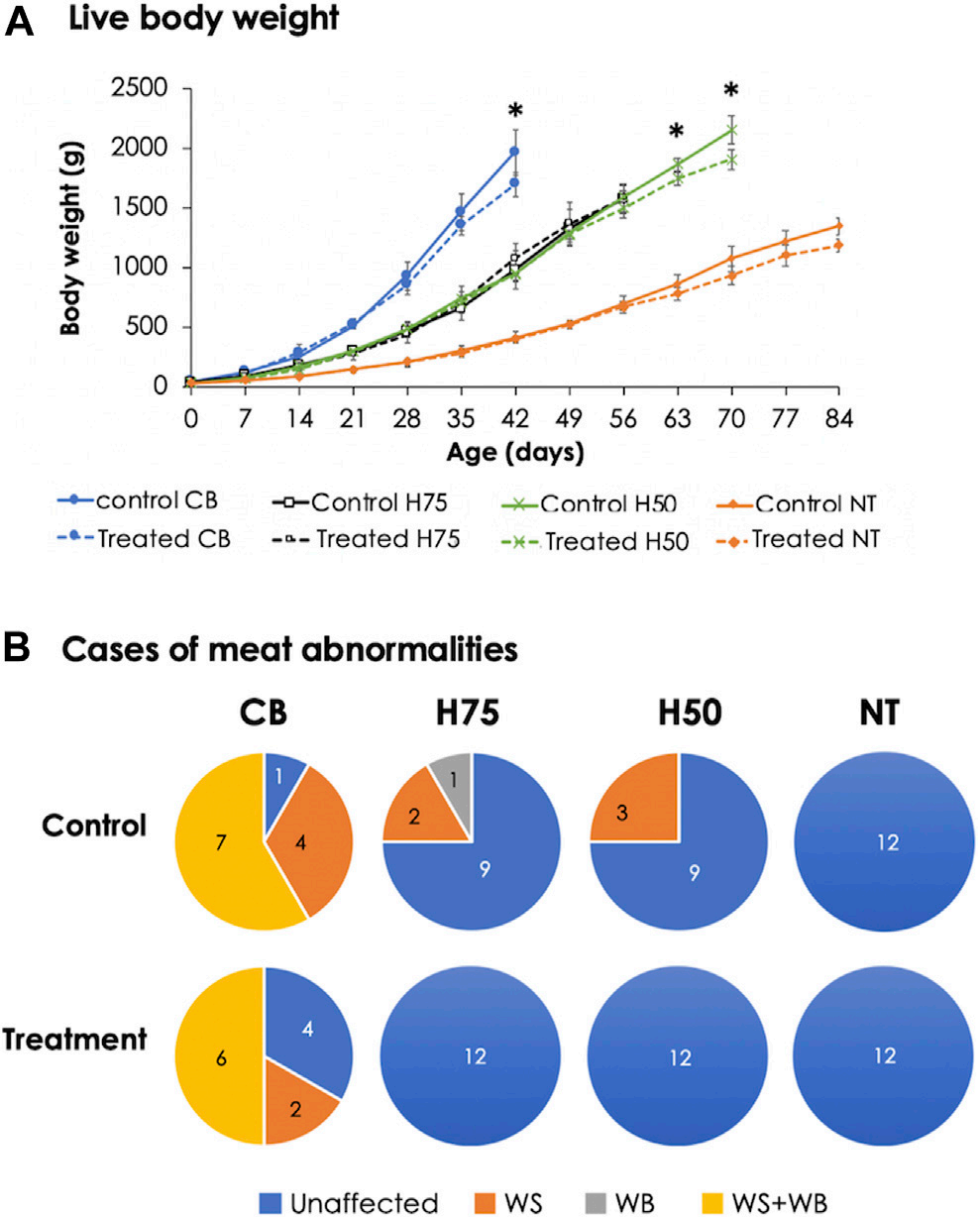
Live body weight and cases of meat abnormalities in the chickens from different breeds. The birds were reared at a constant temperature (control, 26°C) or received heat challenge (treatment, 35°C, 6 h daily) for 20 days before reaching their market ages. **(A)** Markers and error bars depict average and standard deviation of live body weight (*n* = 12 per group), respectively. **(B)** Venn diagrams illustrate occurrence of growth-related myopathies, including white striping (WS) and wooden breast (WB), in breast muscles (*n* = 12 per group). CB: commercial broilers; H75: crossbreeds, 75% broiler background and 25% Thai native background; H50: crossbreeds, 50% broiler background and 50% Thai native background; NT: Thai native chicken.

The H50 birds exhibited the greatest live body weight (*p* < 0.05), followed by CB and H75, whereas NT showed the lowest (*p* < 0.05) body weight. In this regard, the different market age of each chicken strain must be emphasized. The CB, H75, H50, and NT chickens were slaughtered at the age of 42, 56, 70, and 84 days, respectively, following their specific market ages. The superior yield of H50 over H75 might be mainly due to the later age of slaughter as both strains exhibited a similar growth rate (Figure 1A). Although the live body weight of H50 birds was greater than that of CB, the average weight of the breast portion collected from CB, particularly the control group, was greater than the others. The results well-agreed with those of previous studies comparing growth performance among the chickens with different growing rates and with the fact that modern broilers have been selected for their growth rate and pectoralis major yield (Zuidhof et al., 2014).

Concerning WS and WB cases (Figure 1B) of 12 control CB samples, only one breast sample exhibited unaffected characteristics, while four showed WS, and the other seven were classified as WS+WB. As for stressed CB, four unaffected, two WS, and six WS+WB breasts were observed. On the other hand, three of 12 birds from the control H75 and control H50 exhibited the abnormalities, whereas no WS or WB cases were observed among the NT chickens. It is worth mentioning that all WS-affected samples were classified as mild lesions (1–40 white lines clearly observed with line thickness < 1 mm), whereas the majority of the WB-affected breasts fell into the category of Grade 1 in which the breasts were hard mainly in the cranial region but flexible otherwise (Tijare et al., 2016). Of seven WS+WB breasts among the control CB samples, two samples were classified as moderate WB lesion (hardness throughout but slightly flexible in the middle) and one sample fell into severe WB severity (extreme hardness throughout the meat). The results strongly supported the influences of genetic selection for growth rate and meat yield on the development of abnormalities (Kuttappan et al., 2012; Pampouille et al., 2018; Lake et al., 2021). The lower number of cases for WS and WB abnormalities in the stressed CB group might be related with restricted growth among the stressed CB birds. This hypothesis was in agreement with previous reports of Kuttappan et al. (2013a) and Malila et al. (2018) that the high degree of WS and WB was significantly related with heavier birds and pectoral muscle yield. In accordance with our current findings, a recent study of Aslam et al. (2021) showed that Arbor Acres broilers received cycle heat stress (32°C, 12 h daily, 21–42 days) exhibited reduced body weight gain with less cases of WS in comparison to the birds raised under control condition (22–24°C).

Focusing on the chemical composition of the breast samples (Table 4), the cyclic heat stress significantly reduced the moisture content in breast samples (*p* < 0.05), whereas breed difference significantly influenced moisture, protein, fat, and ash content (*p* < 0.05). The interaction (*p* < 0.05) between the main effects (i.e., heat stress and breed) was observed for crude protein content. The control CB comprised lower protein than other samples (*p* < 0.05). Focusing on CB breeds, average values of moisture and fat content of the control CB were about 1.5 and 30% greater than those of their stressed counterparts. The results for CB breast were consistent with the characteristics of the breasts affected with growth-related myopathies (Soglia et al., 2016; Cai et al., 2018; Malila et al., 2018; Dalle Zotte et al., 2020; Maharjan et al., 2020; Thanatsang et al., 2020).

**TABLE 4:** Chemical composition (g/100 g sample) of breast samples.

Sample		Moisture	Crude protein	Crude fat	Carbohydrate	Ash
CB	Control	75.87 ± 0.55	20.87^d^ ± 0.84	1.09 ± 0.19	1.01 ± 0.50	1.16 ± 0.12
—	Treatment	74.74 ± 0.22	22.51^c^ ± 0.27	0.76 ± 0.12	0.59 ± 0.27	1.40 ± 0.07
H75	Control	73.85 ± 0.29	23.03^bc^ ± 0.43	0.72 ± 0.30	0.70 ± 0.26	1.69 ± 0.16
—	Treatment	73.96 ± 1.03	23.05^bc^ ± 1.08	0.60 ± 0.17	0.82 ± 0.37	1.57 ± 0.30
H50	Control	74.12 ± 0.41	23.27^abc^ ± 0.34	0.63 ± 0.27	0.47 ± 0.49	1.51 ± 0.13
—	Treatment	73.25 ± 0.87	24.12^a^ ± 0.57	0.45 ± 0.13	0.45 ± 0.20	1.74 ± 0.20
NT	Control	73.31 ± 0.40	23.91^ab^ ± 0.60	0.28 ± 0.08	0.57 ± 0.21	1.94 ± 0.08
—	Treatment	73.39 ± 0.21	23.63^ab^ ± 0.34	0.42 ± 0.13	0.71 ± 0.32	1.85 ± 0.02
Main effects	—	—	—	—	—	—
Stress	Control	74.29 ± 1.06	22.77 ± 1.29	0.68 ± 0.36	0.69 ± 0.41	1.57 ± 0.32
—	Treatment	73.89 ± 0.87	23.33 ± 0.85	0.56 ± 0.18	0.64 ± 0.30	1.64 ± 0.24
Breed	CB	75.31^a^ ± 0.72	21.69^b^ ± 1.05	0.93^a^ ± 0.23	0.80 ± 0.43	1.28^c^ ± 0.16
—	H75	73.91^b^ ± 0.70	23.04^a^ ± 0.76	0.66^b^ ± 0.24	0.76 ± 0.30	1.63^b^ ± 0.23
—	H50	73.69^b^ ± 0.78	23.69^a^ ± 0.63	0.54 ^bc^ ± 0.22	0.46 ± 0.35	1.62^b^ ± 0.20
—	NT	73.35^b^ ± 0.30	23.77^a^ ± 0.48	0.35^c^ ± 0.12	0.64 ± 0.26	1.89^a^ ± 0.07
*p*-value	—	—	—	—	—	—
Stress	—	0.03	0.02	0.08	0.71	0.25
Breed	—	<0.0001	<0.0001	<0.0001	0.23	<0.0001
Interaction	—	0.08	0.02	0.10	0.38	0.07

Mean ± standard deviation (*n* = 12).

^a, b, c, d^Significant differences due to different treatment groups or different breeds (*p* < 0.05).

It is worth mentioning here that the temperature for control samples in this study was out of the optimum range (21–22°C) recommended for growing commercial broilers. However, as global ambient temperature continues to rise, it is difficult, particularly in tropical and subtropical regions, even for the houses equipped with an evaporative cooling facility, to maintain an optimum range during the day. The average temperature of 24–26°C or even higher was frequently observed in the rearing houses (Tirawattanawanich et al., 2011). We observed that in some recent studies, the thermoneutral condition approximately at 24–26°C was also used (Sohail et al., 2012; Duangjinda et al., 2017; Xu et al., 2018).

### Histological Findings

**FIGURE 2:**
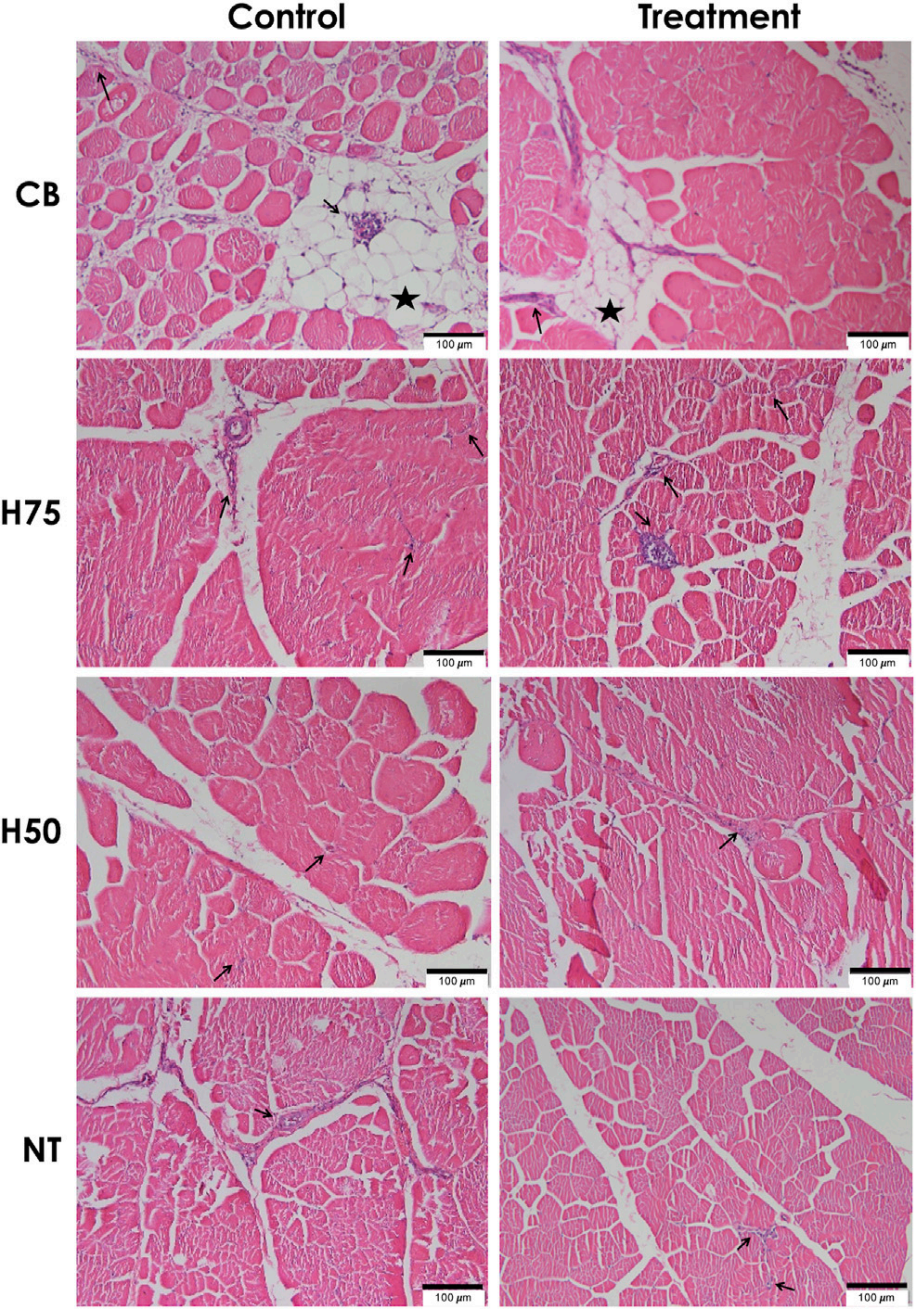
Histological findings for hematoxylin and eosin (H&E)–stained pectoralis major muscle sections. Examples of microscopic images (scale bar of 100 μm) of H&E–stained pectoralis major muscles collected from different chicken breeds. The birds were reared at a constant temperature (control, 26°C) or received heat challenge (treatment, 35°C, 6 h daily) for 20 days before reaching their market ages. CB: commercial broilers; H75: crossbreeds, 75% broiler background and 25% Thai native background; H50: crossbreeds, 50% broiler background and 50% Thai native background; NT: Thai native chickens. Arrows and stars indicate accumulation of inflammatory cells and adipose tissue, respectively.

**FIGURE 3:**
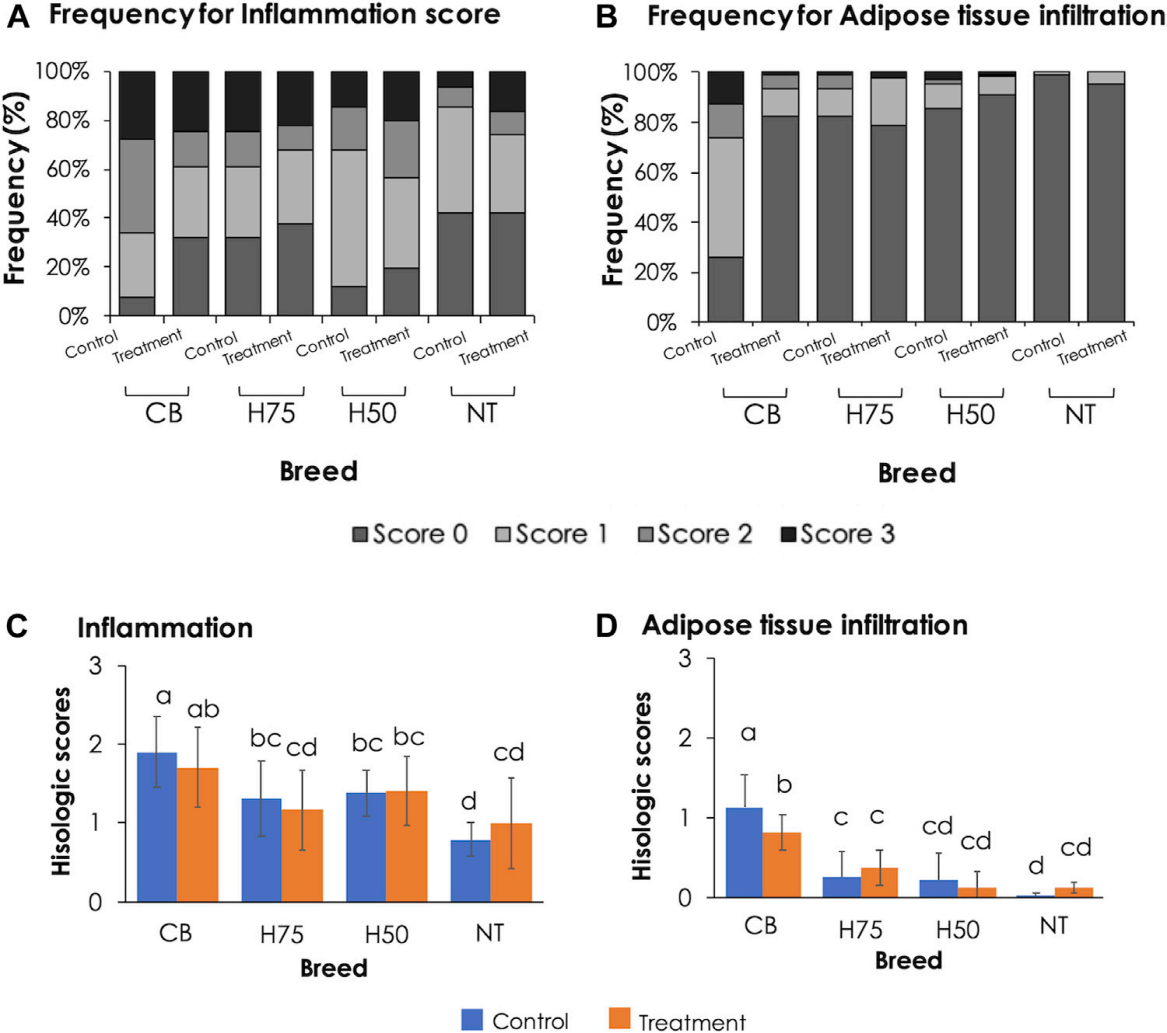
Bar graphs indicating frequency and average histological lesion scores. **(A,B)** Frequency, expressed in percentage, of each histological score observed in pectoralis major muscles of each treatment group. **(C,D)** Average histological scores. Bars and error bars depict average and standard errors in the scores, respectively. CB: commercial broilers; H75: crossbreeds, 75% broiler background and 25% Thai native background; H50: crossbreeds, 50% broiler background and 50% Thai native background; NT: Thai native chickens. Different letters above bars denote statistical significance (*p* < 0.05).

The examples of histological findings of the pectoralis major muscles of each sample group are illustrated in Figure 2. Based on histological scores (Figure 3), the control CB samples frequently received score 2 for inflammation, with less samples receiving score 0 (Figure 3A). Hence, the average score for inflammation (Figure 3C) of the control CB was greater (*p* < 0.05) than that of the samples from the other strains, but the average scores for such histological characteristics were not significantly different from that of the stressed CB (*p* ≥ 0.05). The average scores for inflammation were least pronounced in the control NT, of which the average scores did not significantly differ from stressed NT and stressed H75.

Concerning adipose tissue infiltration (Figure 3B), the majority of the control CB received score 1, while the majority of the other groups, particularly NT samples, was scored 0 for adipose tissue infiltration. The average score (Figure 3D) was the most pronounced in control CB, followed by the stressed CB. The results indicated the significant inflammation and adipose infiltration in the pectoral muscle of the control CB at a greater extent in comparison to the other strains. The histopathological appearance was in correspondence with the abnormal histological characteristics of WS and WB consistently reported in commercial broilers (Kuttappan et al., 2013b; Russo et al., 2015; Sihvo et al., 2017; Prisco et al., 2021).

A previous study of Adu-Asiamah et al. (2021) demonstrated that local Chinese broilers exposed to an acute temperature rise from 30 to 35°C for 8 h at the age of 56 days exhibited infiltrations of fat tissues and inflammatory cells along with mild fibrosis and degenerated muscle fibers in the breast muscle. In this study, although the average histological scores of H75, H50, and NT followed the trend reported in the previous studies, no significant effects were observed, which again supported the ability of those strains to adapt under the current challenge. The discrepancy between our results and the findings of Adu-Asiamah et al. (2021) might be explained by the different thermal intensity between the studies. On the contrary, the CB chickens exposed to the thermal stress were affected with adipose infiltration at a lesser extent than control CB. The current results were in congruence with the report of Aslam et al. (2021) in which less incidence and severity of myodegeneration, inflammatory and lipid infiltration, and fibrosis were observed in the pectoralis major muscle collected from Arbor Acres Plus broilers exposed to cyclic heat stress compared to the birds reared under thermoneutral condition.

### Absolute Gene Expression

**FIGURE 4:**
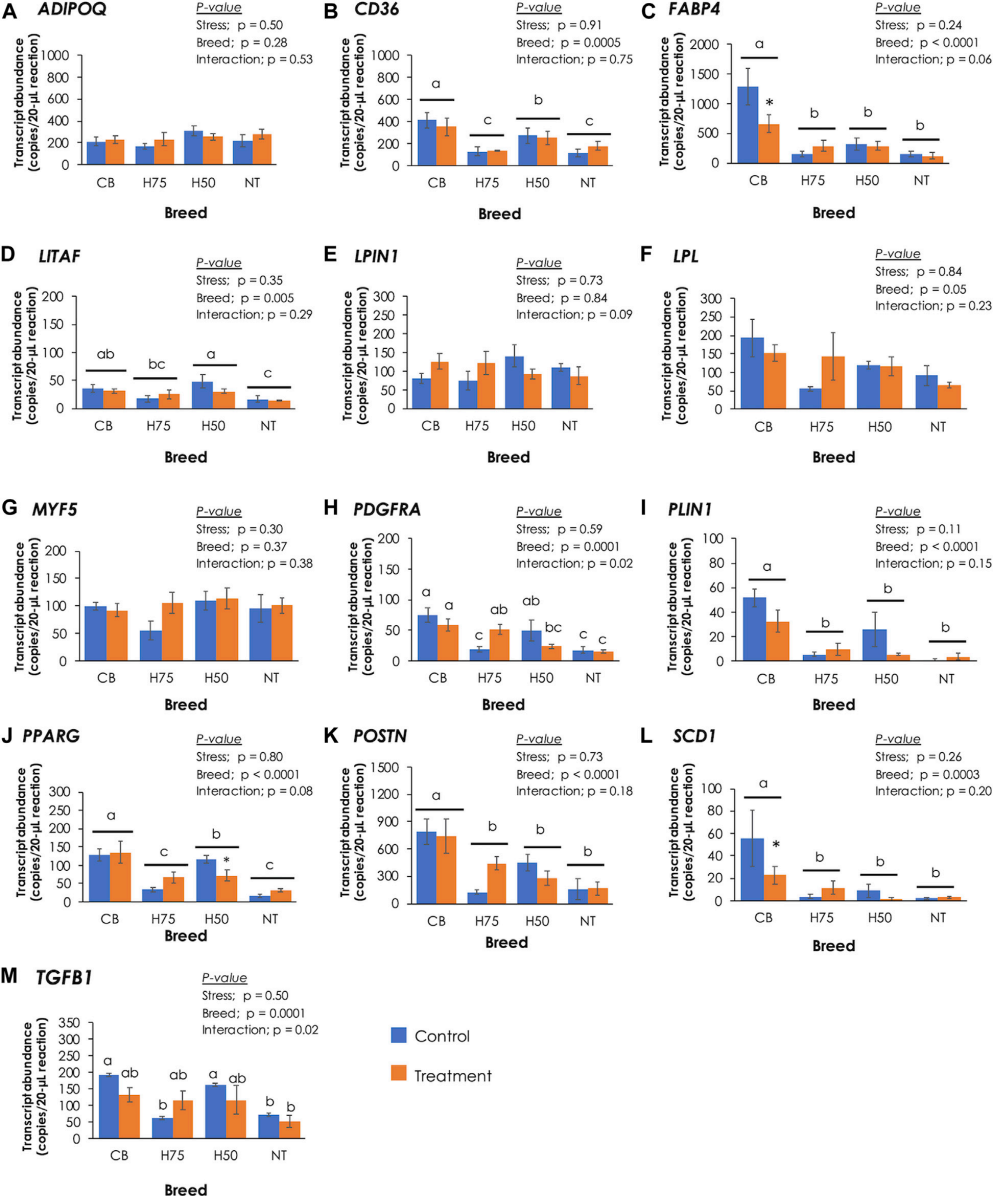
Absolute transcript abundance of 13 target genes associated with lipid metabolisms and muscle injury in the pectoralis major muscle of chickens from different breeds. The birds were reared at a constant temperature (control, 26°C) or received heat challenge (treatment, 35°C, 6 h daily) for 20 days before reaching their market ages. Bars and error bars depict average and standard error in copies per 20-µL reaction, respectively. CB: commercial broilers; H75: crossbreeds, 75% broiler background and 25% Thai native background; H50: crossbreeds, 50% broiler background and 50% Thai native background; NT: Thai native chicken. Different letters above individual bars denote statistical significance among different treatment groups (*p* < 0.05). Different letters above horizontal lines indicate significance due to different breeds (*p* < 0.05). Asterisks indicate the reduced expression of particular genes in stressed samples compared to their control counterparts (*p* < 0.05).

To further elucidate the molecular events associated with histological characteristics, absolute expressions of 13 target genes were examined (Figure 4). Concerning the main effects (heat stress and breed), no significant effects of the cyclic heat stress on transcript abundance of the tested genes were detected (*p* ≥ 0.05). The significant impacts of breed on the expression of *CD36*, *FABP4*, *LITAF*, *PDGFRA*, *PLIN1*, *PPARG*, *POSTN*, *SCD1*, and *TGFB1* were observed (*p* < 0.05). The interaction (*p* < 0.05) between the two main effects was observed for *TGFB1*. The absolute expression levels of those genes, except for *LITAF*, were positively correlated (*p* < 0.05) with histological scores and the occurrence of WS and WB abnormalities (Table 5). Positive correlation (*p* < 0.05) between *LITAF* abundance was detected with adipose tissue infiltration and WS incidence. In addition, the *LPL* expression level was found to be positively correlated (*p* < 0.05) with histological scores for adipose tissue infiltration and WS and WB abnormalities.

**TABLE 5:** Correlation coefficient between histological scores, white striping (WS) and wooden breast (WB) abnormalities, and absolute transcript abundance.

	Pearson’s correlation coefficient		Spearman’s rank correlation coefficient^1^	
	Inflammation	Adipose tissue infiltration	WS	WB
Inflammation	na	na	0.45^*^	0.36
Adipose tissue infiltration	na	na	0.46^*^	0.47**
WS	na	na	na	0.67***
WB	na	na	0.67***	na
*ADIPOQ*	−0.08	−0.12	0.03	0.01
*CD36*	0.43*	0.56**	0.66***	0.51**
*FABP4*	0.57**	0.76***	0.61**	0.51**
*LITAF*	0.29	0.37^*^	0.45*	0.31
*LPIN1*	−0.19	−0.17	0.14	0.03
*LPL*	0.27	0.40^*^	0.51*	0.48**
*MYF5*	−0.18	−0.03	0.37	0.30
*PDGFRA*	0.36*	0.73***	0.51**	0.47**
*PLIN1*	0.61**	0.81***	0.57**	0.47**
*PPARG*	0.49**	0.67***	0.52**	0.43*
*POSTN*	0.38^*^	0.66***	0.44*	0.48**
*SCD1*	0.51**	0.62**	0.54**	0.47**
*TGFB1*	0.47**	0.54**	0.54**	0.42*

*p < 0.05, **p < 0.01, ***p < 0.0001, na = not applicable.

The proteins encoded by *ADIPOQ*, *CD36*, *FABP4*, *LPIN1*, *LPL*, *PLIN1*, *PPARG*, and *SCD1* play crucial roles in lipid metabolism. Adiponectin, encoded by *ADIPOQ*, regulates lipid metabolism by promoting transport of fatty acids into muscle cells and by activities and expression of several enzymes involved in β-oxidation. Adiponectin also promotes accumulation of triglycerides in adipocytes (Nguyen, 2020). Lipoprotein lipase, encoded by *LPL*, catalyzes the rate-limiting step in hydrolysis of plasma lipoprotein triglycerides to nonesterified fatty acids for further utilization of tissues, including re-esterification for storage within tissues (Mead et al., 2022). On the other hand, *LPIN1-*encoding enzyme participates in adipogenesis. *CD36* and *FABP4* encode long-chain fatty acid transport proteins, facilitating the uptake of free fatty acids across sarcolemma into skeletal muscle cells and mitochondria (Stahl et al., 2001). *PLIN1* encodes perilipin-1 which coats on lipid droplets and controls adipocyte triglyceride storage and lipolysis (Sun et al., 2019). *SCD1* encodes stearoyl-CoA desaturase, an integral membrane protein of the endoplasmic reticulum, which catalyzes the formation of monounsaturated fatty acids from saturated fatty acids (Heinemann and Ozols, 2003). In a previous study of Liu et al. (2019), pectoralis major muscle samples were collected from Jingxing-Huang female broilers, divided into two groups based on triglyceride (TG) content, and further submitted to RNA-seq. A transcriptome analysis between the two groups (i.e., high TG vs. low TG) revealed increased transcript abundance of *CD36*, *SCD1*, *PPARG*, *ADIPOQ*, and *LPL* in the high TG samples compared to the low TG ones. Those differentially expressed genes were mapped into the PPARγ signaling pathway, suggesting the crucial role of the PPARγ signaling pathway in lipid deposition in the chicken breast muscle with different TG content. Therefore, the differential expression of *CD36*, *FABP4*, *PLIN1*, *PPARG*, and *SCD1* identified in this study indicated the differences in lipid metabolism among the breeds upon exposure of the current cyclic thermal challenge. The altered lipid utilization could be attributed to the dysregulated PPARγ pathway in promoting adipogenesis and enhanced formation of lipid droplets through activity of *PLIN1* (Liu et al., 2019), leading to intramuscular fat deposition in the breasts.

It is worth noting that previous studies usually reported an increased fat content in the stressed broilers (Quinteiro-Filho et al., 2010; Xu et al., 2018; Lu et al., 2019). However, in this study, the trend was in the opposite direction, where stressed CB exhibited lower fat content along with decrease in absolute expression of *CD36* and *FABP4* than those of control CB. Although the actual reason of this discrepancy requires further investigation, it might be reasonable to hypothesize that the birds might be able to adapt to 20 days of cyclic thermal stress (Aengwanich, 2007). In addition, increased abundances of *CD36*, *PPARG*, *FABP4*, and *LPL* were previously found in the WB-affected broiler breast muscle (Abasht et al., 2016; Lake et al., 2019), while Marciano et al. (2021) reported increased *PLIN1* in the breast muscle of Cobb500 affected with WS condition. Papah and Abasht (2019) addressed significant increase in *CD36*, *PLIN1*, *FABP4*, and *LPL* abundance in the WB-affected pectoralis major muscle collected from 3-week-old Ross 708 broilers; however, in 7-week-old broilers, differential expressions of those genes between the affected and unaffected samples were not observed. Papah and Abasht (2019) hypothesized that as the breast muscle of 7-week-old broilers became larger, hypoxic conditions might be more profound and disrupt transcriptions of PPARγ and its targeted genes, shifting gene expression patterns.

It is widely accepted that an abnormal fat deposition in the skeletal muscle is associated with impaired muscle regeneration, especially in the breast muscle affected by WS. Upon muscle injury, satellite cells are activated and enter cell cycle for muscle regeneration. As myogenic factor 5 (Myf5), encoded by *MYF5*, play roles in activating quiescent satellite cells into proliferation, differential expression of *MYF5* (Figure 4G) among the current samples in correspondence with histological lesions was initially anticipated. However, no significant changes of *MYF5* were found in this study (*p* ≥ 0.05). Although further investigation remained to be elucidated to obtain full comprehension regarding the findings, our data agreed with the previous study of Praud et al. (2020) in which no differences in *MYF5* expression were addressed among slow-growing chickens and broilers exhibited either normal characteristics, WS, WB, or WS/WB abnormalities.

Apart from the adipogenic fate of satellite cells, intramuscular fat in the pectoralis major muscle may also be contributed by activities of fibro-adipogenic progenitors (FAPs). Recently identified by Uezumi et al. (2010), these multi-potent progenitors are localized in the interstitial area of the skeletal muscle and play important roles in muscle repair. During the early phase of muscle regeneration, FAPs are activated and differentiated into adipocytes and collagens to provide a transient support for satellite cell differentiation. FAPs are tightly regulated through TNF-α–induced apoptosis (Lemos et al., 2015). Under abnormal muscle regeneration, an excessive TGF-β could inhibit TNF-α–meditated FAP apoptosis (Pagano et al., 2019) along with imbalanced lipid storage and utilization (Lukjanenko et al., 2013), leading to fat deposition in the skeletal muscle. Overproduction of TGF-β has been observed in the injured skeletal muscle, and its expression is positively correlated with the differentiation fate of FAPs into adipogenic cells (Lukjanenko et al., 2013; Pagano et al., 2019) and fibrogenic cells (Davies et al., 2016; Song et al., 2017; Juban et al., 2018). In this study, absolute transcript abundances of *LITAF* (Figure 4D) and *TGFB1* (Figure 4M) in CB and H50 were greater (*p* < 0.05) than those of NT samples, while the expression levels of those genes in other samples were at intermediate levels. In agreement with the present study, upregulated *TGFB1* and *LITAF* in breast muscle of commercial broilers affected with growth-related myopathies were consistently addressed (Mutryn et al., 2015; Velleman and Clark, 2015; Marchesi et al., 2019; Praud et al., 2020; Prisco et al., 2021; Xing et al., 2021). The role of *LITAF*-encoded protein in regulating the transcription of TNF-α in inflammatory response was previously demonstrated by Hong et al. (2006). *PDGFRA* encodes a cell surface tyrosine kinase receptor for members of the platelet-derived growth factor family which is crucially required for normal development of several cells and organs, including connective tissue (Horikawa et al., 2016). Uezumi et al. (2010) demonstrated that the adipogenic fate of FAPs depended greatly on the muscle microenvironment, and only PDGFRA-positive FAP cells could undergo differentiation into adipocytes in the skeletal muscle. An increased *PDGFRA* abundance was also previously observed in the breast muscle of broilers affected with WB myopathy (Pampouille et al., 2019; Praud et al., 2020). The difference in the expression pattern of *PDGFRA* (Figure 4H) found in this study suggested a potential association between activities of FAPs and intensive histological lesions among the current chicken breast muscle samples.

Although fibrosis was not the main focus in this study, we observed an increased *POSTN* absolute abundance (Figure 4K) in significant association with severity of histological lesions (Table 5) in the current samples. *POSTN-*encoded protein, expressed in connective tissues rich in collagens, is recognized as a key player in regulation of organization of the extracellular matrix and shown to be induced by growth factors and cytokines, particularly TGF-β (González-González and Alonso, 2018). Our previous transcriptome analysis revealed an increased *POSTN* for approximately 2.5-fold in the WB-affected breast muscle of 7-week-old Ross 308 broilers compared to their normal counterparts (Malila et al., 2021b). The upregulated *POSTN* together with *TGFB1* identified in this study may also imply the fibronectin fate of FAPs potentially through the regulation of TGF-β in the control CB.

## Conclusion

In summary, the results indicated that the current cyclic thermal condition significantly reduced live body weight and breast weight of the chickens (*p* < 0.05). As per chemical composition, stressed CB samples exhibited increased protein and ash (*p* < 0.05) content compared with their control counterparts. In addition, infiltration of inflammatory cells and adipose tissues was prevalent in control CB, and the histological scores correlated with the incidence of white striping. Differential absolute transcript abundances of the target genes in the breast muscle samples suggested potential involvement of dysregulated activities of FAPs together with perturbed lipid metabolisms in fat deposition in the CB breast muscle.

## Data Availability

The original contributions presented in the study are included in the article/Supplementary Material, further inquiries can be directed to the corresponding author.
